# Supplementary material for: Climatic Factors Determine the Distribution Patterns of Leaf Nutrient Traits at Large Scales
Source: Plants (Basel). 2022 Aug 21;11(16):2171. doi: 10.3390/plants11162171 (PMC9415000; doi:10.3390/plants11162171)
Supplement: Supplementary file 1 [file plants-11-02171-s001.zip › plants-1880774-supplementary.pdf]

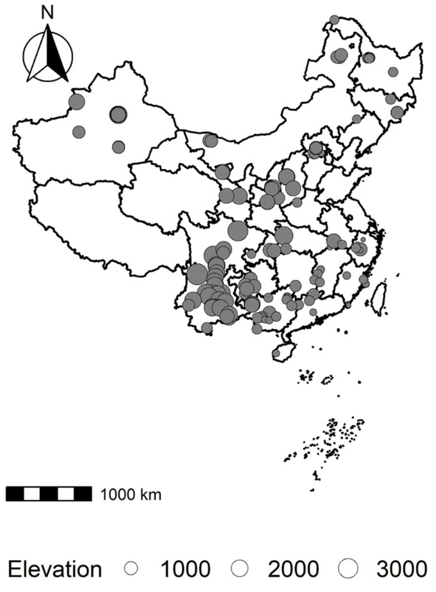

Figure S1. Sample site map of the distribution of the sample sites used for this experimental study in China. The sample sites range from 19.1°N to 53.5°N and 79.7°E to 129.3°E.

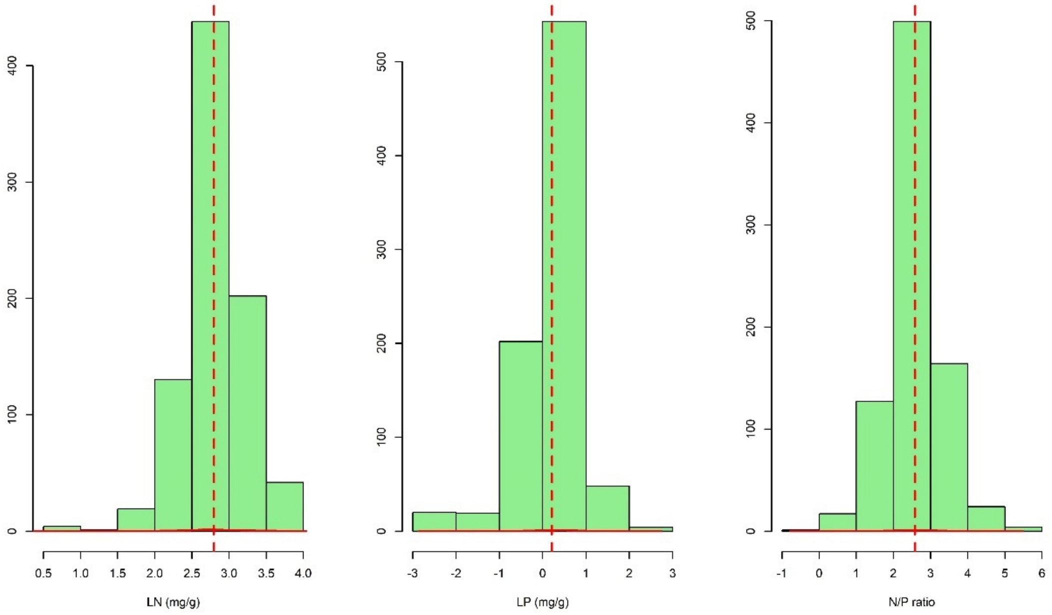

Figure S2. Normal distribution charts for LN, LP, and N/P data.
